# Near-Infrared Time-Resolved Spectroscopy for Assessing Brown Adipose Tissue Density in Humans: A Review

**DOI:** 10.3389/fendo.2020.00261

**Published:** 2020-05-19

**Authors:** Takafumi Hamaoka, Shinsuke Nirengi, Sayuri Fuse, Shiho Amagasa, Ryotaro Kime, Miyuki Kuroiwa, Tasuki Endo, Naoki Sakane, Mami Matsushita, Masayuki Saito, Takeshi Yoneshiro, Yuko Kurosawa

**Affiliations:** ^1^Department of Sports Medicine for Health Promotion, Tokyo Medical University, Tokyo, Japan; ^2^Division of Preventive Medicine, National Hospital Organization Kyoto Medical Center, Clinical Research Institute, Kyoto, Japan; ^3^Dorothy M. Davis Heart and Lung Research Institute, Wexner Medical Center, Columbus, OH, United States; ^4^Department of Preventive Medicine and Public Health, Tokyo Medical University, Tokyo, Japan; ^5^Department of Nutrition, Tenshi College, Sapporo, Japan; ^6^Faculty of Veterinary Medicine, Hokkaido University, Sapporo, Japan; ^7^Diabetes Center, University of California San Francisco, San Francisco, CA, United States

**Keywords:** brown adipose tissue, adaptive thermogenesis, thermogenic food ingredients, androgens, lipid metabolites, seasonal temperature changes, non-invasive, ^18^F-fluorodeoxyglucose–positron emission tomography

## Abstract

Brown adipose tissue (BAT) mediates adaptive thermogenesis upon food intake and cold exposure, thus potentially contributing to the prevention of lifestyle-related diseases. ^18^F-fluorodeoxyglucose (FDG)–positron emission tomography (PET) with computed tomography (CT) (^18^FDG–PET/CT) is a standard method for assessing BAT activity and volume in humans. ^18^FDG–PET/CT has several limitations, including high device cost and ionizing radiation and acute cold exposure necessary to maximally stimulate BAT activity. In contrast, near-infrared spectroscopy (NIRS) has been used for measuring changes in O_2_-dependent light absorption in the tissue in a non-invasive manner, without using radiation. Among NIRS, time-resolved NIRS (NIR_TRS_) can quantify the concentrations of oxygenated and deoxygenated hemoglobin ([oxy-Hb] and [deoxy-Hb], respectively) by emitting ultrashort (100 ps) light pulses and counts photons, which are scattered and absorbed in the tissue. The basis for assessing BAT density (BAT-d) using NIR_TRS_ is that the vascular density in the supraclavicular region, as estimated using Hb concentration, is higher in BAT than in white adipose tissue. In contrast, relatively low-cost continuous wavelength NIRS (NIR_CWS_) is employed for measuring relative changes in oxygenation in tissues. In this review, we provide evidence for the validity of NIR_TRS_ and NIR_CWS_ in estimating human BAT characteristics. The indicators (Ind_NIRS_) examined were [oxy-Hb]_sup_, [deoxy-Hb]_sup_, total hemoglobin [total-Hb]_sup_, Hb O_2_ saturation (StO_2sup_), and reduced scattering coefficient ($\mu_{\text{s sup}}^{\prime }$) in the supraclavicular region, as determined by NIR_TRS_, and relative changes in corresponding parameters, as determined by NIR_CWS_. The evidence comprises the relationships between the Ind_NIRS_ investigated and those determined by ^18^FDG–PET/CT; the correlation between the Ind_NIRS_ and cold-induced thermogenesis; the relationship of the Ind_NIRS_ to parameters measured by ^18^FDG–PET/CT, which responded to seasonal temperature fluctuations; the relationship of the Ind_NIRS_ and plasma lipid metabolites; the analogy of the Ind_NIRS_ to chronological and anthropometric data; and changes in the Ind_NIRS_ following thermogenic food supplementation. The [total-Hb]_sup_ and [oxy-Hb]_sup_ determined by NIR_TRS_, but not parameters determined by NIR_CWS_, exhibited significant correlations with cold-induced thermogenesis parameters and plasma androgens in men in winter or analogies to ^18^FDG–PET. We conclude that NIR_TRS_ can provide useful information for assessing BAT-d in a simple, rapid, non-invasive way, although further validation study is still needed.

## Introduction

Human adipose tissues are of a variety of types, such as white (WAT) and brown adipose tissue (BAT) (1). WAT is capable of depositing extra-energy as triglyceride droplets under conditions where energy intake is greater than its expenditure. In contrast, BAT promotes non-shivering thermogenesis to respond to decreases in core body temperature and, in contrast to WAT, is characterized by an abundance of mitochondria and vasculature. BAT has been extensively investigated in animals, and it has been determined that BAT-specific uncoupling protein (UCP)-1, mainly stimulated upon β_3_-adrenergic activation by cold and/or dietary intervention, enables BAT to dissipate free energy to heat by proton discharge through the inner mitochondrial membrane (2, 3). BAT has drawn renewed attention since 2009, with several papers being published that report the existence of BAT deposits in adult humans (4–7), which had previously been thought to be lost during the process of maturation. Human BAT is reported to be related to lower adiposity [body mass index (BMI), the percentage of whole body fat (%BF), and visceral fat area (VFA)] (6–9) and increased glucose sensitivity (10). In experimental studies, repeated exposure to cold environment enhanced the BAT activity and improved glucose tolerance in obese counterparts (11) and patients with type 2 diabetes mellitus (12) as well as in healthy individuals (9, 13, 14). Thus, increasing BAT activity or volume may aid in combatting obesity and chronic diseases, such as type 2 diabetes mellitus.

It is well-known in humans that BAT can be evaluated by ^18^F-fluorodeoxyglucose (FDG)–positron emission tomography (PET) with computed tomography (CT) (^18^FDG–PET/CT) under cold-stimulated environments (3, 4, 6, 15). However, ^18^FDG–PET/CT has several limitations, including the enormous cost of the device and ionizing radiation exposure, and acute cold exposure—necessary to maximally stimulate BAT activity (16), which make a longitudinal ^18^FDG–PET/CT study difficult and disrupt interventional research, specifically longitudinal ones in humans. Cold exposure is primarily required in ^18^FDG–PET/CT studies to activate human BAT, and various protocols have been applied in the past. While standardized guidelines have recently been proposed, differences between protocols remain a significant obstacle to the comparison of observations from different studies (17).

Other non-invasive technologies have been utilized for evaluating BAT characteristics in humans, such as magnetic resonance imaging (MRI) (18, 19), local skin thermal measurements (19), infrared thermography (20), and contrast ultrasound (21). A recent review on the detection of BAT using these non-invasive technologies can be found elsewhere (22). Regarding MRI technologies, proton-density fat fraction (PDFF) values are widely used to distinguish BAT from WAT (23, 24). However, the PDFF range in the supraclavicular region widely varies among individuals, which makes the differentiation of BAT from WAT difficult, although several new technologies, such as the measurement of T2^*^ relaxation and diffusion-weighted imaging are under investigation (22). The local skin thermal measurements have been used for monitoring cold-induced temperature changes in the supraclavicular skin with infrared thermography (25, 26). However, heat measurements could be influenced by the tissue conductive properties and thickness of the subcutaneous adipose tissue (27), and the individual vasomotor response (28); these evaluation obstacles should be solved in the future.

In addition, near-infrared spectroscopy (NIRS) is a relatively newly introduced methodology to monitor BAT properties (29). The basis for the application of NIRS to evaluate BAT properties is that the microvascular bed, as evaluated by total hemoglobin (Hb) concentration [total-Hb]_sup_ in the supraclavicular region, is more abundant in BAT than in WAT (30). Furthermore, NIR time-resolved spectroscopy (NIR_TRS_) may be used to assess the density of the microvasculature as well as mitochondrial content in BAT by measuring the reduced scattering coefficient ($\mu_{\text{s}}^{\prime }$), which reflects the *in vitro* mitochondrial content (31). BAT is a highly innervated tissue and is also highly perfused when exposed to cold (32). As the concentrations of oxygenated and deoxygenated Hb in the supraclavicular region ([oxy-Hb]_sup_ and [deoxy-Hb]_sup_, respectively) are likely to change (especially [total-Hb]_sup_, which reflects blood volume), it could be a valid measure of BAT vasculature.

The purpose of this article is to provide evidence concerning the ability of NIRS to evaluate BAT characteristics in humans. In this review, we included studies examining BAT characteristics using NIRS in humans: most studies used NIR_TRS_ (29, 33–39), a technology to quantify both absolute tissue absorption and scattering characteristics, while some utilized NIR continuous wave spectroscopy (NIR_CWS_), an inexpensive technology that only provides relative values of tissue oxygenation (32, 40). First, we present how NIRS functions to evaluate tissue oxygenation and blood volume. Then, we provide data indicating whether BAT characteristics can be evaluated using NIR_CWS_. The main body of the paper presents a series of evidence for NIR_TRS_ to assess BAT characteristics. The evidence tested comprises (1) the relationship between parameters determined using NIRS and those measured by ^18^FDG–PET/CT, (2) correlations between the NIRS parameters and cold-induced thermogenesis (CIT), (3) correspondence of the NIRS parameters to those reported using ^18^FDG–PET/CT regarding chronological and anthropometric data, (4) the correspondence between NIRS parameters and those reported with ^18^FDG–PET/CT in response to ambient temperature fluctuations, (5) the relationship between parameters determined using NIRS and plasma lipid metabolites, and (6) changes in NIRS parameters induced by supplementation with evidence-based thermogenic functional ingredients.

## How NIRS Functions as Evaluating Tissue Oxygenation and Blood Volume

NIRS provides non-invasive monitoring of tissue oxygen and Hb dynamics *in vivo* (41–45). NIRS is able to monitor changes in O_2_-dependent light absorption in the heme in the red blood cells circulating in biological tissues (46). There are mainly three types of NIRS devices: NIR_CWS_, NIR_TRS_, phase modulation NIR spectroscopy (NIR_PMS_), etc. (46–48). The most popular NIRS devices use NIR_CWS_ that outputs only the qualitative tissue oxygenation. To calculate the changes in [oxy-Hb], [deoxy-Hb], [total-Hb], and Hb O_2_ saturation (StO_2_) using NIR_CWS_, a combination of multiple-wavelengths can be adopted in accordance with the Beer–Lambert law. The main reason why quantitative data cannot be provided as continuous NIR light path traveled through tissues is unknown (42–45). However, spatially resolved NIRS (a type of NIR_CWS_) is able to provide quantitative values considering several assumptions, although it is still unable to provide the tissue absorption and scattering properties.

On the other hand, NIR_TRS_ and NIR_PMS_ are more accurate, as they can quantify both tissue absorption and scattering characteristics. NIR_TRS_ emits ultrafast (100 ps) light pulses from the skin surface and measures the photon distributions across the biological tissue with a 2- to 4-cm distance from the light emission. NIR_TRS_ is able to quantitatively measure the absorption coefficient (μ_a_), $\mu_{\text{s}}^{\prime }$, and then calculates light path length, tissue [oxy-Hb], [deoxy-Hb], [total-Hb], and StO_2_ (44, 45, 48). The validity of the signal obtained by NIR_CWS_ and NIR_TRS_ has been confirmed in an *in vitro* experiment using highly scattering Intralipid™ (43, 44). Using this system, μ_a_ in the NIR range was found to be strongly correlated with [total-Hb] (43, 48). Furthermore, the study found a significant relationship between $\mu_{\text{s}}^{\prime }$ at 780 nm and the homogenized tissue mitochondrial concentration (31).

## Studies Using NIR_CWS_

Prior to the NIR_TRS_ study on human BAT, one study attempted to correlate oxygen dynamics using NIR_CWS_ and BAT parameters (32). In this cross-sectional study, adult human subjects (25 subjects; 15 women and 10 men; mean age ± SD, 30 ± 7 years) were assigned into high- (BMI, 22.1 ± 3.1) and low-BAT groups (BMI, 24.7 ± 3.9) based on the levels of ^18^F-FDG uptake in the cervical–supraclavicular region. It employed triple-oxygen PET scans (${\text{H}}_{2}^{15}$O, C^15^O, and ^15^O_2_) and daily energy expenditure measurements under resting and mild cold (15.5°C) room conditions for 60 min using indirect calorimetry (32). They used a NIR_CWS_ parameter, adjusted supraclavicular StO_2_ (adjStO_2_), a balance between oxygen supply and uptake. In the high-BAT group, there was a significant negative correlation between oxygen consumption determined by PET scans and adjStO_2_ (*p* = 0.02, *r*^2^ = 0.46) in the supraclavicular region at rest and after the exposure to cold, indicating increased oxygen uptake in highly active BAT (32). However, it detected a limited effect on the difference in adjStO_2_ between the two groups (32). It should be noted that the study presented several limitations to consider when interpreting its results: (1) a non-individualized cooling protocol was used; (2) only one NIR_CWS_ parameter, adjStO_2_, was used in the analysis; and (3) no kinetics data determined by the NIR_CWS_ were provided.

Recently, in young healthy women, a study using the standardized cold exposure aimed to investigate the association between NIR_CWS_ parameters in the supraclavicular and forearm regions and BAT capacity assessed by ^18^FDG–PET/CT (40). Briefly, the subjects arrived at the laboratory (a room temperature of 19.5–20°C) and wore a temperature-controlled water circulation cooling vest for 60 min, and the individual temperature to be exposed was determined, namely at ~4°C above the threshold of shivering, 48–72 h prior to the ^18^FDG–PET/CT measurements. No association was found between any NIR_CWS_ indicators and maximal standardized uptake value (SUV_max_) and mean standardized uptake value (SUV_mean_) of the radioactivity both under thermoneutral and cold conditions. Thus, NIR_CWS_ would not be an appropriate technology to evaluate BAT capacity in this demographic. The lack of significant association between NIR_CWS_ parameters is mainly due to differences in the instrumentation to that used in NIR_TRS_, which provides absolute values for tissue hemodynamics. Furthermore, NIR_CWS_ permits an ~15 mm depth of light penetration at a 30 mm input-output setups (44). However, the mean photon penetration would be deeper (~20 mm at the 30-mm input–output setups) and wider when NIR_TRS_ is used (49), which influences the differences in sensitivity between NIR_CWS_ and NIR_TRS_ with respect to BAT detection. Table 1 shows the relationship between [oxy-Hb]_sup_, [deoxy-Hb]_sup_, [total-Hb]_sup_, StO_2sup_, and adjStO_2sup_ determined by NIR_CWS_ and ^18^FDG–PET/CT parameters (SUV_max_ and SUV_mean_), which have been documented in previous studies (32, 40). The only NIR_CWS_ parameter found to be significantly correlated with a ^18^FDG–PET/CT parameter, cold-induced oxygen uptake by BAT, is adjStO_2_, and only in the high-BAT group.

**Table 1:** Parameters obtained by continuous-wave near-infrared spectroscopy (NIR_CWS_) for evaluating brown adipose tissue characteristics in the supraclavicular and control muscle regions.

					**Correlation (*****r***^****2****^**)**										
					**Supraclavicular region**						**Deltoid (forearm) region**				
**Ref. no**.	**Instrument**	***n***	**Study design**	**Parameters**	**$\mu_{\text{s}}^{\prime }$**	**Oxy-Hb**	**Deoxy-Hb**	**Total-Hb**	**StO_**2**_**	**adjStO_**2**_**	**$\mu_{\text{s}}^{\prime }$**	**Oxy-Hb**	**Deoxy-Hb**	**Total-Hb**	**StO_**2**_**
Muzik et al. (32)	NIR_CWS_	25	Cross-sectional	${\text{VO}}_{2\text{BAT}}{\;}^{\#}$	ND	ND	ND	ND	NM	0.46^*^	ND	ND	ND	ND	NM
Acosta et al. (40)	NIR_CWS_	18	Cross-sectional	SUV_mean_	ND	−0.24	−0.06	−0.24	−0.20	NM	ND	0.01	0.08	0.04	−0.01
				SUV_peak_	ND	−0.06	−0.22	−0.07	−0.04	NM	ND	0.11	0.19^*^	0.15	0.05
				BAT volume	ND	0.00	−0.06	−0.02	−0.02	NM	ND	−0.01	0.12	0.03	−0.06

The correlation coefficients of parameters determined by NIR_CWS_ and the uptake of ^18^F-fluorodeoxy glucose (FDG) are presented under cold-exposed condition.

$\mu_{\text{s}}^{\prime }$, reduced scattering coefficient determined by NIR_TRS_; oxy-Hb, oxygenated hemoglobin (Hb); deoxy-Hb, deoxygenated Hb; total-Hb, total Hb; StO_2_, tissue hemoglobin oxygen saturation; adjStO_2_, adjusted StO_2_ in the supraclavicular region relative to the deltoid muscle; VO_2BAT_, oxygen consumption in BAT; SUV_mean_, the mean standardized uptake value of the radioactivity (SUV) assessed by ^18^FDG–PET/CT; SUV_max_, the maximal SUV assessed by ^18^FDG–PET/CT; BAT volume, evaluated by summating all voxel volume with SUV >2.0 assessed by ^18^FDG–PET/CT; Ref. no., reference numbers are obtained from the list of references in this paper; NM, not mentioned; ND, could not be determined.

^#^Data obtained under thermoneutral conditions.

* *P < 0.05*.

Taken together, the studies using NIR_CWS_ (32, 40) present potential limitations beyond the fact that it cannot evaluate the absorption and scattering properties of the tissue, including that it is more sensitive to changes in the skin blood flow than NIR_TRS_ (32, 40). Therefore, NIR_CWS_ does not seem to be a valid measure for BAT function although emphasis should be placed in the need for further research examining this type of NIRS.

## Studies Using NIR_TRS_

### Correlation Between Parameters Determined by NIR_TRS_ and ^18^FDG–PET/CT Parameters

It is speculated that, as BAT exhibits higher microvascular density and mitochondrial contents compared to WAT, NIR_TRS_ can be used for assessing the density of microvascular or mitochondrial content in BAT (BAT-d) by measuring [total-Hb]_sup_ and $\mu_{\text{s}}^{\prime }$ in the supraclavicular region ($\mu_{\text{s sup}}^{\prime }$), which reflects the *in vitro* mitochondrial content (31). It may be expected that BAT would exhibit higher values for [total-Hb]_sup_ and $\mu_{\text{s sup}}^{\prime }$ than those exhibited by WAT. As NIR_TRS_ measures the average tissue hemoglobin concentration in a volume of 4 cm^3^ with a 3-cm optode separation (50), the ^18^FDG–PET/CT-determined SUV_mean_ is the most appropriate indicators for comparison to those determined using NIR_TRS_. Although the use of other radiotracer techniques, such as triple-oxygen scans (${\text{H}}_{2}^{15}$O, C^15^O, and ^15^O_2_) would be more appropriate to reflect BAT activity, ^18^FDG–PET/CT has been mostly used for determination of BAT activity owing to routine availability in a clinical science setup. First, 18 healthy male subjects (20.3 ± 1.8 years and BMI of 23.9 ± 3.1 kg/m^2^) were recruited to examine changes in NIR_TRS_ parameters under acute cold environment. The [oxy-Hb]_sup_, [deoxy-Hb]_sup_, [total-Hb]_sup_, StO_2sup_, and $\mu_{\text{s sup}}^{\prime }$ were compared between a room temperature under thermoneutral conditions and cold conditions (19°C) for 2 h. As there was no change in the [total-Hb]_sup_ and $\mu_{\text{s sup}}^{\prime }$ at 19°C compared to baseline conditions measured at 27°C, NIR_TRS_ can be used without the necessity of cold exposure (29). Second, to test this hypothesis, [total-Hb]_sup_ and $\mu_{\text{s sup}}^{\prime }$ were compared to the SUV_mean_ assessed by ^18^FDG–PET/CT (29) in a cross-sectional design. Twenty-nine healthy male subjects (23.3 ± 2.2 years and BMI of 21.6 ± 1.8 kg/m^2^) were recruited to evaluate the relationship between SUV_mean_ and NIR_TRS_ parameters. The results demonstrated that [total-Hb]_sup_ and $\mu_{\text{s sup}}^{\prime }$ under warm environment is significantly correlated with the SUV_mean_ under cold environment but only in the supraclavicular fossa, a region of BAT located (29). Other parameters, except adjStO_2_ specifically in the supraclavicular region, also showed significant correlation with SUV_max_ and SUV_mean_ under cold environment (Table 2). Collectively, the [total-Hb]_sup_, [oxy-Hb]_sup_, and [deoxy-Hb]_sup_ show significant correlations to BAT activity determined by ^18^FDG–PET/CT. StO_2sup_, however, proved to be inferior, and adjStO_2_ was completely insensitive to changes in BAT activity (Table 2).

**Table 2:** Parameters for evaluating brown adipose tissue characteristics using near-infrared time-resolved spectroscopy (NIR_TRS_) in the supraclavicular and control muscle regions.

					**Correlation (*****r***^**2**^**)**										
					**Supraclavicular region**						**Deltoid (forearm) region**				
**Ref. no**.	**Instrument**	***n***	**Study design**	**Parameters**	**$\mu_{\text{s}}^{\prime }$**	**Oxy-Hb**	**Deoxy-Hb**	**Total-Hb**	**StO_**2**_**	**AdjStO_**2**_**	**$\mu_{\text{s}}^{\prime }$**	**Oxy-Hb**	**Deoxy-Hb**	**Total-Hb**	**StO_**2**_**
Nirengi et al. (29)	NIR_TRS_	18	Cross-sectional	SUV_mean_	0.41^*^	0.52^*^	0.48^*^	0.53^*^	0.14^*^	0.07	0.08	0.25	0.21	0.20	0.08
				SUV_max_	0.44^*^	0.52^*^	0.49^*^	0.53^*^	0.18^*^	0.08	0.04	0.27	0.23	0.12	0.05

The correlation coefficients of tissue-oxygenated hemoglobin (oxy-Hb), deoxygenated Hb (deoxy-Hb), total Hb (total-Hb), tissue Hb oxygen saturation (StO_2_,), and optical scattering parameters as determined by NIR_TRS_ under thermoneutral conditions and the uptake of ^18^F-fluorodeoxy glucose (FDG) or cold-induced thermogenesis are presented.

$\mu_{\text{s}}^{\prime }$, reduced scattering coefficient determined by NIR_TRS_; adjusted StO_2_ in the supraclavicular region relative to the deltoid muscle; SUV_mean_, the mean standardized uptake value of the radioactivity (SUV) assessed by ^18^FDG–PET/CT; SUV_max_, the maximal SUV assessed by ^18^FDG–PET/CT; BAT volume, evaluated by summating all voxel volume with SUV >2.0 assessed by ^18^FDG–PET/CT; Ref. no., reference number is obtained from the list of references in this paper.

* *P < 0.05*.

A 2-h cold exposure doubles the BAT blood flow (32), which appeared to be inconsistent with our observations. In an attempt to interpret this apparent discrepancy, we speculated that NIR_TRS_ parameters are susceptible to the change in the volume but less sensitive to the change in the flow. The blood flow can be calculated by multiplying the blood flow velocity by the cross-sectional area of the vessel (the volume). There is presently a lack of NIR_TRS_-derived data concerning blood flow in BAT. Alternatively, while muscle blood flow increases by some 10-fold during peak exercise (51, 52), [total-Hb], an indicator of blood volume, monitored by NIR_TRS_ elevates only 1.1-fold (43). Thus, the change in blood volume measurable by NIR_TRS_ is marginal compared to increases in blood flow velocity during metabolic activation. Collectively, both $\mu_{\text{s sup}}^{\prime }$ and [total-Hb]_sup_ were evaluated using the NIR_TRS_ technique can be applied to assess BAT-d in humans and are equivalent to the active BAT intensity or the BAT volume, as measured by ^18^FDG–PET/CT under cold environment (29). Usually, to assign participants into high-BAT (BAT [+]) and low-BAT (BAT [–]) groups, a cutoff value of 2.0 for SUV_mean_ is applied. The accuracy of [total-Hb]_sup_ or $\mu_{\text{s sup}}^{\prime }$ in representing BAT activity was analyzed. Accordingly, the area under the receiver operating characteristic (ROC) curve was determined by SUV_mean_ of 2.0 nearest to (0, 1) for $\mu_{\text{s sup}}^{\prime }$ and [total-Hb]_sup_ (29). When 74.0 μM or 6.8 cm^−1^ was selected as the cutoff value, meaning that [total-Hb]_sup_ or $\mu_{\text{s sup}}^{\prime }$ larger than 74.0 μM or 6.8 cm^−1^, respectively, are regarded as BAT [+], ROC analysis yields results that are very good when compared to SUV_mean_ (29).

### Correlation Between Parameters Determined by NIR_TRS_ and CIT

It is well-documented in rodents that the upregulation of the UCP-1 in brown adipocytes upon cold increases whole body oxygen consumption, termed as CIT. Although several authors have shown that CIT does not always reflect BAT activity (53), that the contribution of BAT thermogenesis to CIT is marginal (~10 kcal/day when maximally activated) (54), and no correlation is found between BAT and CIT (55), the magnitude of CIT is related to the amount of BAT activity and/or volume (9, 56–59). Thus, having already observed a significant correlation between NIR_TRS_ parameters and SUV_mean_ assessed by ^18^FDG–PET/CT (29) in humans, the validity of NIR_TRS_ parameters were further examined by comparing [total-Hb]_sup_ and $\mu_{\text{s sup}}^{\prime }$ to CIT in healthy individuals [age of 20.0 (median), 19.0 (the first quartile), and 21.0 (the third quartile) year; BMI of 24.2 (21.6, 25.7) kg/m^2^] with [total-Hb]_sup_ of 50–125 μM in a cross-sectional study (37). The participants sat for 20 min at 27°C with a light clothing, and NIR_TRS_ measurements were conducted for 5 min after fasting for 6–12 h. Then, the participants were tested at room temperature of 19°C for 2 h with their feet intermittently placed on a cloth-wrapped ice for ~4 min every 5 min (9). A significant correlation was found between [total-Hb]_sup_, [oxy-Hb]_sup_, or [deoxy-Hb]_sup_ only under thermoneutral conditions and CIT, but not between adjStO_2_ or $\mu_{\text{s sup}}^{\prime }$ and CIT (37). In contrast, previous studies reported a significant correlation in the supraclavicular region between the adjStO_2_ and oxygen consumption by BAT under cold environment and between $\mu_{\text{s sup}}^{\prime }$ and SUV_max_ and SUV_mean_ (29, 32). It is noted that a personalized cooling protocol may be a better procedure to induce a CIT response personalized to each individual (60). Collectively, although the [total-Hb]_sup_, [oxy-Hb]_sup_, and [deoxy-Hb]_sup_ are markers for BAT activity as evaluated by CIT, the adjStO_2_ and $\mu_{\text{s sup}}^{\prime }$ become less sensitive to CIT (Table 3).

**Table 3:** Relationship between parameters determined by near-infrared time-resolved spectroscopy (NIR_TRS_) under thermoneutral or cold condition and pulmonary oxygen uptake during cold exposure.

					**Correlation (*****r***^**2**^**)**									
					**Supraclavicular region**					**Deltoid (forearm) region**				
**Ref. no**.	**Instrument**	***n***	**Study design**	**Parameters**	**$\mu_{\text{s}}^{\prime }$**	**Oxy-Hb**	**Deoxy-Hb**	**Total-Hb**	**StO_**2**_**	**$\mu_{\text{s}}^{\prime }$**	**Oxy-Hb**	**Deoxy-Hb**	**Total-Hb**	**StO_**2**_**
Nirengi et al. (37)	NIR_TRS_	18	Cross-sectional	CIT 27°C	0.00	0.38^*^	0.49^*^	0.41^*^	0.14	0.09	0.06	0.04	0.06	0.04
				CIT 19°C	0.08	0.24^*^	0.16	0.23^*^	0.01	0.15	0.02	0.09	0.04	0.06

Results of NIR_TRS_ parameters [tissue-oxygenated hemoglobin (oxy-Hb), deoxygenated Hb (deoxy-Hb), total Hb (total-Hb), tissue Hb oxygen saturation (StO_2_,), and optical scattering parameters] and cold-induced thermogenesis (CIT) for healthy men under thermoneutral (27°C) or cold condition (19°C) are presented.

$\mu_{\text{s}}^{\prime }$, reduced scattering coefficient determined by NIR_TRS_.

* *P < 0.05*.

### Relationship Between NIR_TRS_ Parameters in the Supraclavicular Region and Chronological and Anthropometric Data

^18^FDG–PET/CT studies have revealed that a significant relationship exists between BAT activity and chronological and anthropometrical parameters (4, 5, 29, 56). Cold-stimulated ^18^FDG–PET/CT studies have shown that BAT activity negatively associated with age, sex, BMI, %BF mass, and VFA, and also that BAT was a significant independent determinant of glucose and HbA1c levels, after adjustment for age, sex, and body adiposity (10, 56).

**Figure 1:**
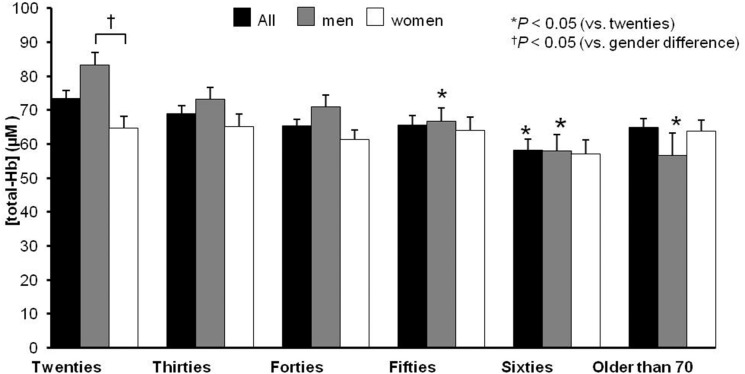
Chronological and sex differences in terms of the concentration of the supraclavicular total hemoglobin [total-Hb], an indicator of brown adipose tissue (BAT). The supraclavicular [total-Hb] potentially containing BAT. The BAT-positive rate (SUV_max_ > 2.0) is indicated in the bottom of the figure based on previous studies (29, 33). The values are presented as means ± standard error (SE), adjusting for body mass index, body fat ratio, and visceral fat area. ^©^SPIE. Reproduced by permission of the publisher. Adopted from reference (33).

A cross-sectional study using NIR_TRS_ demonstrated that [total-Hb]_sup_ under warm environment was negatively associated with age and body adiposity in 413 Japanese individuals [a median age of 43.0 (33.0–58.0, interquartile range) years, BMI of 22.5 (20.7–24.5) kg/m^2^, and %BF of 26.8% (20.6–32.3%)] in winter (33) (Figure 1). With the exception of participants in their 20s, there were no sex-related differences in [total-Hb]_sup_ among the groups tested (Figure 1). Multivariate analyses revealed that the %BF and VFA were significantly negatively correlated with [total-Hb]_sup_ (33). The observation of the study was analogous to data acquired using ^18^FDG–PET/CT, indicating the usefulness of the parameter [total-Hb]_sup_. In contrast, $\mu_{\text{s}}^{\prime }$ was found to be significantly negatively correlated with some of the anthropometrical parameters. Together, the [oxy-Hb] _sup_ and [deoxy-Hb] _sup_ displayed similar accuracy to the [total-Hb]_sup_ for detecting relationships with chronological and anthropometric data (Table 4).

**Table 4:** Relationship between parameters in the supraclavicular region obtained by near-infrared time-resolved spectroscopy (NIR_TRS_) and anthropometric and body composition parameters.

					**Correlation (*****r***^****2****^**)**					
					**Supraclavicular region**					
**Ref. no**.	**Instrument**	***n***	**Study design**	**Parameters**	**$\mu_{\text{s}}^{\prime }$**	**Oxy-Hb**	**Deoxy-Hb**	**Total-Hb**	**StO_**2**_**	**AdjStO_**2**_**
Fuse et al. (33)	NIR_TRS_	413	Cross-sectional	Age	0.00	0.07^*^	0.04^*^	0.06^*^	0.02^*^	NM
				BMI	0.02^*^	0.11^*^	0.12^*^	0.12^*^	0.00	NM
				%body fat	0.00	0.16^*^	0.10^*^	0.15^*^	0.02^*^	NM
				Visceral fat area	0.01^*^	0.13^*^	0.12^*^	0.14^*^	0.00	NM

The correlation coefficients of brown adipose tissue (BAT)-related parameters [tissue oxygenated hemoglobin (oxy-Hb), deoxygenated Hb (deoxy-Hb), total Hb (total-Hb), tissue Hb oxygen saturation (StO_2_,), and optical scattering parameters] as determined by NIR_TRS_ and body composition and anthropometric parameters are presented.

$\mu_{\text{s}}^{\prime }$, reduced scattering coefficient determined by NIR_TRS_; adjStO_2_, adjusted StO_2_ in the supraclavicular region relative to the deltoid muscle; BMI, body mass index; Ref. no., reference number is obtained from the list of references in this paper; NM, not mentioned.

* *P < 0.05*.

### Changes in NIR_TRS_ Parameters in the Supraclavicular Region in Response to Ambient Temperature Fluctuations

BAT increases in winter according to ^18^FDG–PET/CT studies (4, 56–59). However, one study reported that early winter showed higher BAT activity than late winter or early spring (61). A cross-sectional study (35) reported that [total-Hb]_sup_ was higher in winter than in summer. It has also been reported that a lower average ambient temperature during the 4–6 weeks before the measurement day increases [total-Hb]_sup_ (33). This finding is consistent with previous findings reporting that, while BAT activity rose during the winter, a few months are needed for the increase in BAT activity after a decrease in the air temperature (58). A longitudinal study using the same healthy subjects (men/women, 35/23; mean age, 37.4 years; mean BMI, 22.5 kg/m^2^; BAT positive rate, 48%) in summer and winter under thermoneutral conditions revealed a significant increase in [total-Hb]_sup_, but not in the reference region or in the $\mu_{\text{s}}^{\prime }$ from any regions (37). It is unclear why $\mu_{\text{s sup}}^{\prime }$ region did not change between summer and winter. However, it is speculated that if triglyceride droplet in the supraclavicular area decreases in winter owing to the increase in BAT (or beiging), the $\mu_{\text{s}}^{\prime }$ should decrease because WAT (triglyceride droplet) obtains high scattering characteristics (62). Thus, even increasing the mitochondria content (the increase in $\mu_{\text{s}}^{\prime }$) would offset the decrease in WAT (the decrease in $\mu_{\text{s}}^{\prime }$), indicating that [total-Hb]_sup_ may be a better indicator of BAT activity than $\mu_{\text{s sup}}^{\prime }$. We demonstrated seasonal changes in other NIR_TRS_ parameters, which supplement previously published findings in Table 5 (35, 37). The [oxy-Hb]_sup_ and the [total-Hb]_sup_ obtain similar tendency for monitoring seasonal fluctuations in BAT-d. In winter, the decrease in the deltoid [deoxy-Hb] demonstrated that muscle metabolism blunted in winter and that this is less reliable than [oxy-Hb]_sup_ or [total-Hb]_sup_ (Table 5).

**Table 5:** Changes in parameters obtained by near-infrared time-resolved spectroscopy (NIR_TRS_) by seasonal temperature changes.

					**Change by supplementation or season (%)**										
					**Supraclavicular region**						**Deltoid region**				
**Ref. no**.	**Instrument**	***n***	**Study design**	**Modulation**	**$\mu_{\text{s}}^{\prime }$**	**Oxy-Hb**	**Deoxy-Hb**	**Total-Hb**	**StO_**2**_**	**AdjStO_**2**_**	**$\mu_{\text{s}}^{\prime }$**	**Oxy-Hb**	**Deoxy-Hb**	**Total-Hb**	**StO_**2**_**
Nirengi et al. (35)	NIR_TRS_	40	Cross-sectional	Season	10.0	31.0^*^	9.2	23.7^*^	5.9^*^	5.1^*^	7.2	28.7	23.7	27.2	0.5
Nirengi et al. (37)	NIR_TRS_	58	Longitudinal	Season	−4.1	16.5^*^	5.7	12.9^*^	2.8^*^	1.5^*^	−0.3	−0.5	−4.5^*^	−3.0	1.3^*^

The percentage changes in the NIR_TRS_ parameters [tissue oxygenated hemoglobin (oxy-Hb), deoxygenated Hb (deoxy-Hb), total Hb (total-Hb), tissue Hb oxygen saturation (StO_2_,), and optical scattering parameters] are presented due to seasonal temperature fluctuations.

$\mu_{\text{s}}^{\prime }$, reduced scattering coefficient determined by NIR_TRS_; adjStO_2_, adjusted StO_2_ in the supraclavicular region relative to the deltoid muscle; NIR_CWS_, NIR continuous-wave spectroscopy; Ref. no., reference numbers are obtained from the list of references in this paper.

* *P < 0.05*.

Collectively, the [oxy-Hb]_sup_, [total-Hb]_sup_, StO_2sup_, and adjStO_2_ can detect seasonal fluctuations of BAT-d, which is consistent with the findings of previous ^18^FDG–PET/CT studies (4, 56–59).

### Correlation Between NIRS Parameters in the Supraclavicular Region and Lipid Metabolites

Finding blood biomolecules correlated with BAT characteristics would permit us to advance human BAT studies because PET/CT studies may be difficult owing to ionizing radiation and cold exposures. Studies on lipidomic profiles have clarified BAT and WAT characteristics according to muscle contractions or cold environment (11, 12, 14, 16). BAT characteristics are related to unique profiles of lipid metabolites, such as the concentration of lysophosphatidylcholine-acyl (LysoPC-acyl) C16:0 in humans (63), and the concentration of phosphatidylethanolamine (PE) in the BAT and WAT was decreased in high-fat diet-fed mice (14). The relationships have been examined in the winter and summer between [total-Hb]_sup_, a parameter for evaluating BAT-d, measured using NIR_TRS_ and plasma lipids in humans (38). Healthy volunteers with [total-Hb]_sup_ values over 74.0 μM (high BAT-d) were studied (*n* = 23) and control volunteers with lower [total-Hb]_sup_ values <70.0 μM (low BAT-d) (*n* = 23) were tested. Ninety-two plasma samples were examined (23 men and 23 women, aged 21–55; BMI, 21.9 ± 3.0 kg/m^2^, %BF 23.3 ± 8.0%), in summer and winter. Using liquid chromatography-time-of-flight-mass spectrometry, plasma lipid profiles were determined. The [total-Hb]_sup_ was determined as a parameter of BAT-d using NIR_TRS_ under thermoneutral conditions. Body composition parameters, such as %BF and VFA, were examined. Univariate and multivariate regression analyses were used to determine factors affecting [total-Hb]_sup_. In men, there were 37 metabolites showing positive correlations and 20 metabolites showing negative correlations (*P* < 0.05) with [total-Hb]_sup_, respectively. After the *Q* values were obtained by correcting false discovery rate, only androgens (testosterone, androstanedione, dehydroandrosterone, dehydroepiandrosterone, or epitestosterone) showed a significant (*Q* < 0.05) positive correlation with [total-Hb]_sup_ in men in winter. Multivariate regression analysis revealed that [total-Hb]_sup_ showed a significant correlation with androgens in men and VFA in women in winter. Notably, the [total-Hb]_sup_ showed a significant relationship with androgens in winter in men but did not with any body-composition characteristics, such as whole body and visceral adiposity, which are generally associated with [total-Hb]_sup_. Although androgens deteriorated the BAT capacity *in vitro* (64), testosterone induced a preferable effect on BAT activity, body adiposity, and energy expenditure in animal models (65–67). Thus, BAT characteristics might be predicted by measuring plasma androgens as a biomarker in men in the winter. However, further detailed research is needed to discover biomarkers that predict BAT in women.

### Changes in NIRS Parameters in the Supraclavicular Region by Thermogenic Functional Ingredients

Recent studies have demonstrated that BAT can improve health status and has a protective effect on lifestyle-related diseases (4–11, 13, 14). Consequently, research has been focused on finding methods for effectively enhancing BAT activity and/or mass (9, 11–14). Developed strategies include cold acclimation (9, 11–14) and acute treatment of β_3_-adrenergic receptor (AR) agonists in humans (68). However, cold exposure intervention would not be easy to apply to daily life (9), and β3-AR agonists may elicit unpreferable influence, including a risk for hypertension and increased susceptibility to arterial sclerosis (68). Recent investigations have revealed the mechanisms underlying the effects of thermogenic food ingredients. Pathways involved include the transient receptor potential channels (TRP)-BAT axis, a site of adaptive thermogenesis evoked by β-adrenoceptor activation (69). The TRP-BAT axis comprises the activation of cold-sensitive TRP channels located in peripheral tissues, such as the skin and intestines. The activation of TRP channels results in the signal delivery through the afferent nerve to the hypothalamus, which then evokes sympathetic nerve activation within BAT. This causes norepinephrine (NE) release, initiating β-adrenergic tracts to brown adipocytes and eliciting UCP1 upregulation and adaptive thermogenesis (69). In contrast to cold exposure intervention, functional food ingredients may be easily incorporated into daily life. This has been confirmed in animals and humans and includes capsinoids as TRP vanilloid 1 agonists, catechins as TRPA1/V1 agonists, and so on (70). Furthermore, they have the benefit of having no apparent side effects (9, 34, 36, 69, 70).

**Figure 2:**
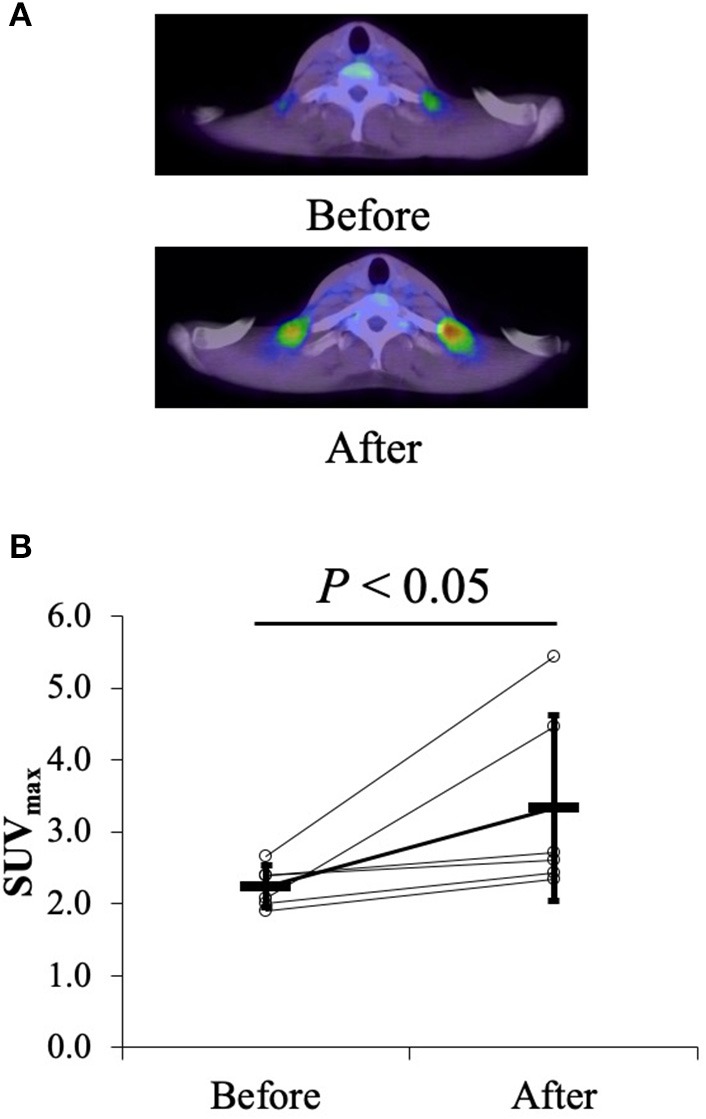
**(A)** Typical images of the uptake of ^18^F-fluorodeoxy glucose (FDG) (mean standardized uptake value) before and after the 6-weeks capsiate supplementation in the supraclavicular region. **(B)** Maximal standardized uptake value (SUV_max_) pre- and post-supplementation. ^©^SPIE. Reproduced by permission of the publisher. Adopted from reference (34).

**Figure 3:**
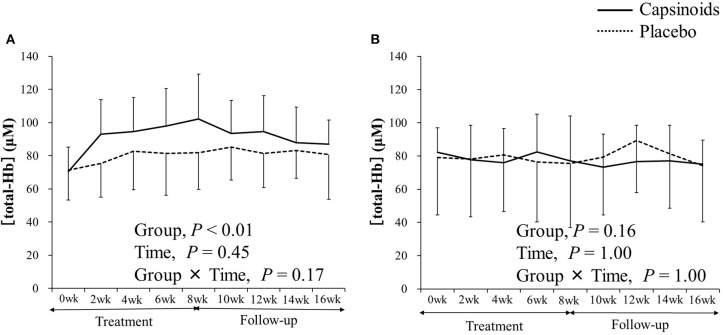
The concentration of total hemoglobin [total-Hb], an indicator of brown adipose tissue (BAT) in **(A)** the supraclavicular fossa potentially containing BAT and **(B)** the deltoid, a control region. Adopted from Nirengi et al. (34). ^©^SPIE. Reproduced by permission of the publisher.

Among thermogenic food ingredients, substances, such as capsiate are known to increase BAT activity (9, 70). Previously, the effect of capsiate on [total-Hb]_sup_, determined by the NIR_TRS_, was examined (34). Twenty healthy individuals [capsiate group (*n* = 10) vs. placebo group (*n* = 10), 20.7 ± 1.2 years vs. 20.9 ± 0.9 years; BMI, 21.4 ± 1.8 vs. 21.9 ± 1.0 kg/m^2^; %BF, 21.3 ± 7.6% vs. 22.9 ± 8.7%] were supplemented either with capsiate (9 mg/day) daily for 8 weeks or a placebo in a paralleled, double-blind manner, and [total-Hb]_sup_ was measured during the treatment period, and for an 8-weeks follow-up period under thermoneutral conditions (34). The study also measured BAT activity with ^18^FDG–PET/CT under cold-exposure conditions as previously reported (29). This was only done twice (not every 2 weeks), pre- and post-supplementation, to reduce participant exposure to ionizing radiation. The study demonstrated a parallel change in BAT-d (+46.4%, *P* < 0.05) pre- and post-supplementation, evaluated as [total-Hb]_sup_, or as BAT activity (+48.8%, *P* < 0.05) evaluated as the SUV_max_, a parameter of the BAT capacity, by ^18^FDG–PET/CT, after the supplementation of thermogenic capsiate (Figures 2, 3). During the 8-weeks follow-up period, the [total-Hb]_sup_ decreased both in the capsiate and placebo groups; the decrease was greater in the capsiate group (albeit not significantly, *P* = 0.07) compared to that of the placebo group.

Previous studies examined whether a catechins-rich green tea extract increases energy consumption in humans (71–74). Animal studies have shown that catechin intake increases BAT, the effects of which were abolished when the β-blocker was administrated (75, 76). Thus, we used NIR_TRS_ under thermoneutral conditions to test the effect of sustained catechin-rich ingredient (540 mg/day) intake on [total-Hb]_sup_ and investigated potential associations between changes in [total-Hb]_sup_ and body adiposity in 22 healthy women college students [catechin group (*n* = 10) vs. placebo group (*n* = 11), 21.1 ± 2.0 years vs. 20.5 ± 2.1 years; BMI, 21.1 ± 1.3 vs. 20.9 ± 1.6 kg/m^2^; %BF, 24.0 ± 3.5% vs. 25.8 ± 7.6%] (36). That study revealed that the [total-Hb]_sup_ was elevated by 19% in the catechin group only after 12 weeks (36). As for the $\mu_{\text{s}}^{\prime }$, which was not documented in the previous study, it did not change during catechin ingestion. There was a significant negative relationship between the enhancement in [total-Hb]_sup_ and the decrease in extramyocellular lipids, an indicator for possible insulin insensitivity (77), in the vastus lateralis muscle determined by proton-magnetic resonance spectroscopy (*r* = −0.66, *P* < 0.05).

After further analysis, some of which has not been documented in the previous study (34), capsiate supplementation was shown to cause a significant increase in [total-Hb]_sup_, [oxy-Hb]_sup_, and [deoxy-Hb]_sup_ and, upon its withdrawal, a decrease in [total-Hb]_sup_, [oxy-Hb]_sup_, StO_2sup_, and adjStO_2_ (Table 6). Similarly, by the catechins intervention, the [oxy-Hb]_sup_, [deoxy-Hb]_sup_, StO_2sup_, and adjStO_2_ obtain the same result as the [total-Hb]_sup_ for assessing increases in BAT-d (36). Collectively, studies into functional ingredient supplementation using NIR_TRS_ suggest that [oxy-Hb]_sup_ and [total-Hb]_sup_ are particularly suitable for the evaluation of BAT-d in intervention studies where the use of ^18^FDG–PET/CT is not applicable (Table 6).

**Table 6:** Changes in parameters obtained by near-infrared time-resolved spectroscopy (NIR_TRS_) for evaluating changes in brown adipose tissue characteristics by the supplementation with thermogenic ingredients.

					**Change by supplementation or season (%)**										
					**Supraclavicular region**						**Deltoid region**				
**Ref. no**.	**Instrument**	***n***	**Study design**	**Modulation**	**$\mu_{\text{s}}^{\prime }$**	**Oxy-Hb**	**Deoxy-Hb**	**Total-Hb**	**StO_**2**_**	**AdjStO_**2**_**	**$\mu_{\text{s}}^{\prime }$**	**Oxy-Hb**	**Deoxy-Hb**	**Total-Hb**	**StO_**2**_**
Nirengi et al. (34)	NIR_TRS_	20	Capsinoid	8 weeks on	9.3	49.5^*^	41.0^*^	46.4^*^	1.80	0.80	9.7^*^	−6.0	−7.5	−6.7	1.1
				8 weeks washout	11.1	−16.4^*^	−2.5	−12.5^*^	−4.8^*^	−4.2^*^	0.6	−2.1	1.3	−1.4	−0.7
Nirengi et al. (36)	NIR_TRS_	22	Catechins	12 weeks on	1.8	15.6^*^	35.1^*^	21.5^*^	−4.8^*^	−10.3^*^	−9.3	12.4	−8.5	4.0	7.3

The percentage changes in the NIR_TRS_ parameters [tissue oxygenated hemoglobin (oxy-Hb), deoxygenated Hb (deoxy-Hb), total Hb (total-Hb), tissue Hb oxygen saturation (StO_2_,), and optical scattering parameters] are presented due to the supplementation with thermogenic ingredients.

$\mu_{\text{s}}^{\prime }$, reduced scattering coefficient determined by NIR_TRS_; adjStO_2_, adjusted StO_2_ in the supraclavicular region relative to the deltoid muscle; NIR_CWS_, NIR continuous-wave spectroscopy; Ref. no., reference numbers are obtained from the list of references in this paper.

* *P < 0.05*.

### Limitations and Perspectives

The studies using NIRS contain several limitations. Several optical issues should be considered, as the multilayer, inhomogeneous tissue property created by skin, adipose tissue, and muscle may affect *in vivo* tissue scattering and absorption characteristics and modulation of optical path. In a study (39), the optical characteristics in the deltoid, abdominal, and supraclavicular regions were tested using NIR_TRS_. The results indicate that there are unique region-specific relationships between [total-Hb] and $\mu_{\text{s}}^{\prime }$, suggesting that examining the [total-Hb]–$\mu_{\text{s}}^{\prime }$ relationship is a practical way to distinguish BAT from other tissues. It could be noted that due to the nature of optical measurements, the placement of the optodes for the NIR_TRS_ must be always secure and in the same area, especially during longitudinal studies. Although NIR_TRS_ is able to quantify tissue oxygen dynamics, the values are affected by optical characteristics underlying subcutaneous adipose tissue in the supraclavicular region, which varies depending on the body composition of subjects, thereby influencing NIR_TRS_ measurements. The reason is that the values obtained is diluted by the lower [Hb] in the subcutaneous adipose tissue (78). The [total-Hb]_sup_ values can be recalculated by considering the thickness of the adipose layer (79).

As no change in the [total-Hb]_sup_ and $\mu_{\text{s sup}}^{\prime }$ was observed during 2-h conditions at 19°C compared to baseline conditions at 27°C (29), NIR_TRS_ cannot detect changes in BAT characteristics responding to an acute cold exposure in nature because NIR_TRS_ is insensitive to changes in the blood flow (33, 35–37). However, a newly developed NIR_TRS_ system contains six wavelengths (760, 800, 830, 908, 936, and 976 nm), of which the latter three wavelengths are adopted to detect optical characteristics of lipids and water (80). This system could provide information on the changes in tissue water and lipid content in response to acute interventions, such as experimental cold exposure, which cannot be obtained using the conventional three-wavelength NIR_TRS_ system. The new six-wavelength NIR_TRS_ system could contribute further insight on the chronic as well as acute responsiveness of BAT metabolism in humans.

Finally, future studies should obtain further evidence to validate BAT evaluation using NIR_TRS_ because^18^FDG–PET/CT measurements include several limitations. BAT mainly consumes intracellular lipids, as well as plasma non-esterified fatty acids and those derived from lipoproteins—whereas ^18^FDG–PET/CT measures a glucose analog. The lack of standardization when quantifying BAT by ^18^FDG–PET/CT is also a problem. Thus, additional experiments to reach this conclusion are required, such as (1) examining whether NIR_TRS_ parameters actually represent the *in vivo* mitochondrial density of BAT, or are related to molecules implicated in the vascularization and thermogenesis of BAT [e.g., vascular endothelial-cell growth factor (VEGF), UCP-1, peroxisome proliferator-activated receptor γ coactivator 1-α (PGC1-α)]. This could be carried out by taking human biopsies from the supraclavicular area and examining the relationship between NIR_TRS_ parameters and the molecular signature of this tissue; (2) using other radiotracers beyond ^18^F-FDG, such as ^15^O, ${\text{H}}_{2}^{15}$O, C^15^O, or ^11^C-acetate, which will allow to measure the real oxygen consumption, tissue perfusion, and metabolic activity of human BAT and which are more likely to represent the thermogenic nature or activity of this tissue than ^18^F-FDG; (3) carrying out studies where the kinetics of NIR_TRS_ are related to the kinetics of the metabolic activity of BAT (dynamic PET/CT); (4) using different cooling protocols, aiming to standardize the cooling stress to which individuals are submitted (avoiding potential biases in individual BAT activation); and (5) performing reliability studies to examine whether NIR_TRS_ measures can be replicated in the short and long term.

## Conclusion

Correlation coefficients are presented for parameters determined by NIRS and ^18^FDG–PET/CT, CIT, or anthropometric and body composition parameters. Significant correlations were found between [total-Hb]_sup_, [oxy-Hb]_sup_, [deoxy-Hb]_sup_, $\mu_{\text{s sup}}^{\prime }$, StO_2sup_, or adjStO_2_ and ^18^FDG–PET/CT indicators; between [total-Hb]_sup_, [oxy-Hb]_sup_, or [deoxy-Hb]_sup_ and CIT; and between [total-Hb]_sup_, [oxy-Hb]_sup_, or [deoxy-Hb]_sup_ and anthropometric and body composition indicators. The percentage changes in NIR_TRS_ parameters as a consequence of either seasonal temperature fluctuations or dietary supplementation with thermogenic ingredients are presented. Seasonal temperature fluctuations influenced [total-Hb]_sup_, [oxy-Hb]_sup_, StO_2sup_, and adjStO_2_. Studies on thermogenic capsinoid or catechin supplementation revealed a significant increase in [total-Hb]_sup_, [oxy-Hb]_sup_, and [deoxy-Hb]_sup_. Upon withdrawal of these supplements, a decrease in [total-Hb]_sup_, [oxy-Hb]_sup_, StO_2sup_, and adjStO_2_ was seen. Recently, androgens were found to show a significant positive correlation with [total-Hb]_sup_ only in men in winter. Thus, BAT characteristics might be predicted by measuring plasma androgens as a biomarker in men in the winter.

We conclude that NIR_TRS_ would be a useful non-invasive technology for assessing BAT-d, although further validation is still needed. Among the parameters evaluated by NIR_TRS_, the [oxy-Hb]_sup_ as well as [total-Hb]_sup_ would be applicable to assessing BAT characteristics in both cross-sectional and interventional studies.

## Author Contributions

TH, SN, SF, and YK collected the relevant literature and wrote the manuscript. SA, RK, MK, and TE assisted in illustrations, formatting, and collection of literature. NS, MM, MS, and TY coordinated and edited the relevant discussion on PET/CT measurements and BAT.

## Conflict of Interest

The authors declare that the research was conducted in the absence of any commercial or financial relationships that could be construed as a potential conflict of interest. The reviewer FA declared a past co-authorship with one of the authors TH to the handling editor.

## Abbreviations

adjStO_2_: adjusted supraclavicular StO_2_
AR: β3-adrenergic receptor
BAT: brown adipose tissue
BAT-d: vascular or mitochondrial density in BAT
BMI: body mass index
CIT: cold-induced thermogenesis
CT: computed tomography
deoxy-Hb: deoxygenated Hb
FDG: ^18^F-fluorodeoxyglucose
Hb: hemoglobin
NE: norepinephrine
NIRS: near-infrared spectroscopy
NIR_CWS_: NIR continuous-wave spectroscopy
NIR_TRS_: NIR time-resolved spectroscopy
oxy-Hb: oxygenated Hb
StO_2_: Hb O_2_ saturation
PET: positron emission tomography
ROC: receiver operating characteristic
total-Hb: total hemoglobin
TRP: transient receptor potential channels
SUV_max_: maximal standardized uptake value
SUV_mean_: mean standardized uptake value
WAT: white adipose tissue
μ_a_: absorption coefficient
$\mu_{\text{s}}^{\prime }$: reduced scattering coefficient.
